# Supplementary material for: Repeated multi-domain cognitive training prevents cognitive decline, anxiety and amyloid pathology found in a mouse model of Alzheimer disease
Source: Commun Biol. 2023 Nov 10;6:1145. doi: 10.1038/s42003-023-05506-6 (PMC10638434; doi:10.1038/s42003-023-05506-6)
Supplement: Supplementary file 2 — Supplementary Figures [file 42003_2023_5506_MOESM2_ESM.pdf]

Title: Repeated multi-domain cognitive training prevents cognitive decline, anxiety and amyloid pathology found in a mouse model of Alzheimer disease

Authors: Jogender Mehla[1#], Scott H. Deibel[1*], Hadil Karem[1], Nancy S. Hong[1], Shakhawat R Hossain[1], Sean G. Lacoursiere[1], Robert J. Sutherland[1], Majid H. Mohajerani[1*] Robert J. McDonald[1*]

[1]Canadian Centre for Behavioural Neuroscience, University of Lethbridge, Lethbridge, Alberta, Canada.

[*] Majid H. Mohajerani (mohajerani@uleth.ca) and Robert J. McDonald (r.mcdonald@uleth.ca) are corresponding authors, contributed equally to the manuscript, and share the senior author position for this paper.

[#]Current address: Department of Neurological Surgery, Washington University School of Medicine, St. Louis, MO, 63110, USA.

*Current address: Department of Psychology, University of New Brunswick, POB 4400, Fredericton, NB, E3B 3A1, Canada.

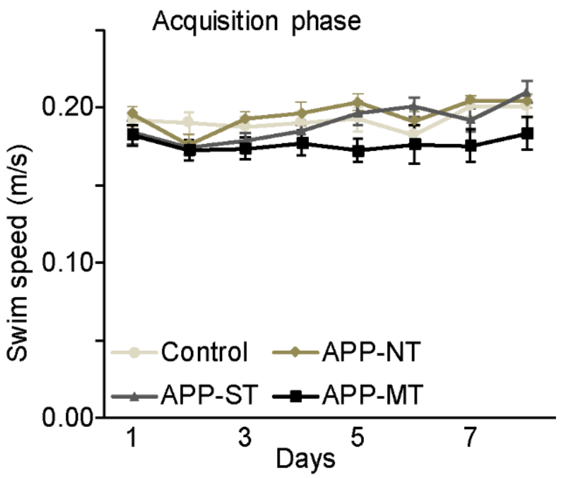

Supplementary Fig. 1 Effects of cognitive training on swim speed of 12 months old mice in the MWM. Data is presented as mean ± SEM. Control group- C57BL/6. APP-NT group- APP$^{NL-G-F/NL-G-F}$ with no training. APP-MT group- APP$^{NL-G-F/NL-G-F}$ mice exposed to multi-domain cognitive training (MT). APP-ST group- APP$^{NL-G-F/NL-G-F}$ mice exposed to single-domain cognitive training (ST).

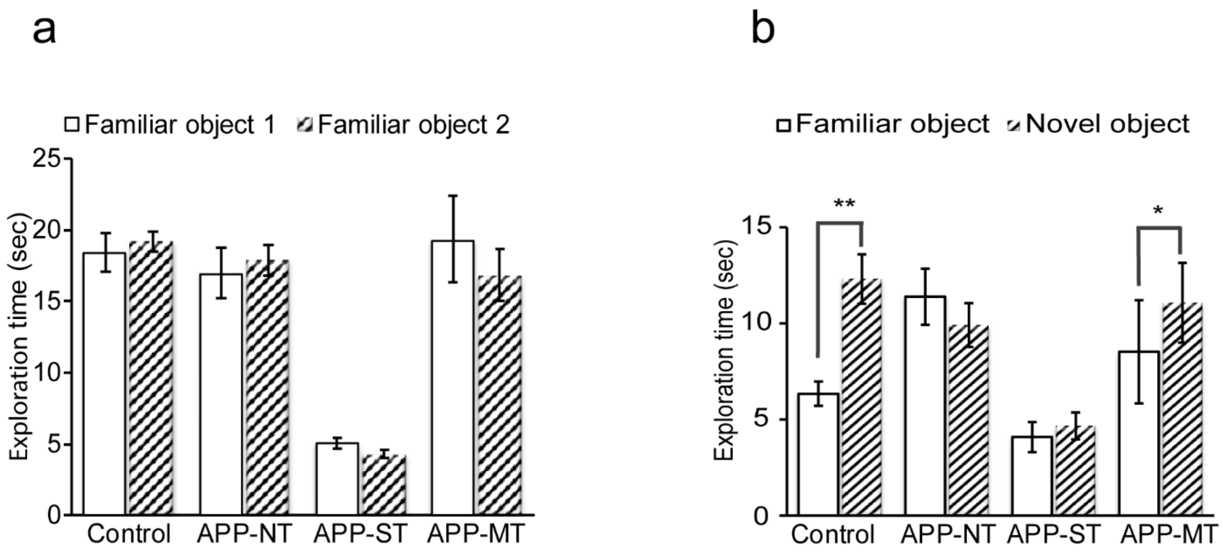

Supplementary Fig. 2 Effects of cognitive training on exploration time of 12 months old mice in object recognition test. **a** Exploration time for familiar objects during training phase. **b** Exploration time for familiar object and novel object during testing phase. Data is presented as mean ± SEM. *$P < 0.05$, **$P < 0.01$ Familiar vs Novel. Control group- C57BL/6. APP-NT group- $APP^{NL\text{-}G\text{-}F/NL\text{-}G\text{-}F}$ with no training. APP-MT group- $APP^{NL\text{-}G\text{-}F/NL\text{-}G\text{-}F}$ mice exposed to multi-domain cognitive training (MT). APP-ST group- $APP^{NL\text{-}G\text{-}F/NL\text{-}G\text{-}F}$ mice exposed to single-domain cognitive training (ST).
