# Supplementary material for: Repeated multi-domain cognitive training prevents cognitive decline, anxiety and amyloid pathology found in a mouse model of Alzheimer disease
Source: Commun Biol. 2023 Nov 10;6:1145. doi: 10.1038/s42003-023-05506-6 (PMC10638434; doi:10.1038/s42003-023-05506-6)
Supplement: Supplementary file 3 — Description of Additional Supplementary Files [file 42003_2023_5506_MOESM3_ESM.pdf]

Description of Additional Supplementary Files

File name: Supplementary Data 1
Description: The source data for all figures and supplementary figures.
